# Management of soft tissue tumors of the upper extremity: a review

**DOI:** 10.1051/sicotj/2017001

**Published:** 2017-06-28

**Authors:** Kevin T. Jubbal, Gehaan D’Souza, Reid A. Abrams, Anna A. Kulidjian

**Affiliations:** 1 University of California, San Diego School of Medicine 9500 Gilman Drive La Jolla CA 92093 USA; 2 Division of Plastic Surgery, University of California, San Diego 200 W Arbor Drive San Diego CA 92103 USA; 3 Department of Orthopedic Surgery, University of California, San Diego 200 W Arbor Drive San Diego CA 92103 USA

**Keywords:** Upper extremity, Sarcoma, Hand tumor, Limb salvage

## Abstract

*Introduction*: Management of malignant tumors of the hand and wrist is challenging and is generally approached by limb salvage or amputation. With advances in care, amputation has been superseded by limb salvage as the treatment of choice.

*Methods*: A narrative literature review was performed to identify articles on the topic of management of soft tissue tumors of the upper extremity, including surgical management, adjuvant radiation therapy, and chemotherapy.

*Results*: A total of 29 articles were selected. Earlier reports favored radical tumor resection, which often led to amputation, whereas later articles demonstrated limb salvage as the preferential treatment modality.

*Conclusions*: Given the detrimental effects on function and psychologic outcomes, amputation has been superseded by limb salvage in most cases, although it can occasionally be the only option. A variety of adjuvant therapies have been described, including radiation or brachytherapy, chemotherapy, and regional hyperthermia. Radiation treatment, and specifically brachytherapy, is beneficial to select patients. Controversy surrounds chemotherapy in certain subtypes, and regional hyperthermia requires further investigation.

## Introduction

Most soft tissue tumors in the upper extremity are benign, and soft tissue sarcomas are rare in the hand and wrist [1]. The primary goal in treating malignant hand tumors is oncologic cure, and the secondary goal is maintenance of maximum function and appearance [2, 3]. Resection should achieve clear margins, preserve functional length, prevent joint contractures and neuromas, and minimization of morbidity [2]. Traditional treatment has focused on amputation, justified by the notion that overzealous functional preservation would result in compromise of surgical margins and increased risk of recurrence [4]. Amputation provides adequate local control, however it sacrifices function and has significant psychological consequences [5, 6]. Therefore treatment of extremity tumors, including hand tumors, has trended toward limb-sparing surgery combined with radiation therapy [5, 7]. Given the complex anatomy of the hand and wrist, radical tumor resection here often means above-the-elbow amputation, thus highlighting the importance of limb salvage surgery considerations in upper extremity sarcoma [1]. Prior to the 1980s, amputation was the mainstay of treatment given higher recurrence with salvage resection, which has since improved through the implementation of more advanced surgical techniques [8]. Recently, limb salvage has become the preferential treatment over amputation [2] and is now considered the standard of care for soft tissue sarcomas of the extremities [9]. At least 85–95% of soft tissue sarcomas and osteosarcomas are now treated with limb-sparing methods, thus reconstruction has become an area of increasing importance [10, 11].

This article attempts to explore current thinking on treatment of soft tissue sarcomas of the upper extremity and establish an overview of limb salvage techniques.

## Comparison of amputation to limb salvage

Although limb salvage with chemotherapy and radiation is now considered the standard of care, there are still some instances where amputation may remain the best method for cure [2]. Limb salvage is an impractical therapy for larger and more invasive tumors. Often the simplest method in reconstruction with rapid wound healing and least complex coverage is preferred [2]. Amputation is more likely to be appropriate when there is tumor involvement into neurovascular structures, anticipated poor functional outcomes, and patient preference [5]. Some cases would result in improved functional outcomes with amputation and prosthesis compared to limited surgery [5]. In some instances, simple amputation without efforts at complex reconstruction may actually provide the least morbidity and the fastest return to desired functional levels [2]. Amputation is also more appropriate in patients who have had multiple local recurrences in the past [8]. Generally, amputation is more likely to be appropriate for distal lesions in which the limitation to function is relatively reduced [1].

In comparing the two options in terms of the primary goal of oncologic cure, the rate of local recurrence was initially higher in patients who had limb salvage over amputation [12], however survival was equivalent [7, 13]. The initial local control rate seemed improved in amputation, however the overall survival did not improve thus indicating that modern limb salvage is equivalent to amputation in terms of survival [8]. These developments resulted in increased use of limb salvage with increasing reliance on adjuvant therapy [7]. These initially higher recurrence rates with limb salvage compared to amputation were likely due to more primitive techniques of early limb salvage therapy [8]. While amputation still results in lower recurrence rates, the observed benefit diminishes as limb salvage techniques improve. Now, limb salvage is used in the majority of patients with extremity sarcomas without increased rates of recurrence, metastasis, or death compared to amputation [11].

Overall, limb salvage often provides good functional outcome [6]. Further studies have also demonstrated that limited surgery with functional sparing in addition to radiotherapy can provide adequate local control and survival [5]. Local control with limited surgery is highly dependent and significantly improved with adequate surgical resection, such that adjuvant or neoadjuvant therapy does not compensate for inadequate margins [5, 14]. It is also important to remember that radiotherapy carries the risk of complications of nearby structures, thus impairing function, which can be reduced through careful planning [4]. Radiotherapy is associated with improved local control after surgical resection without effect on overall survival [5]. In terms of functional outcomes, some studies have demonstrated that radiotherapy has a detrimental effect in the hand as measured by the grip strength [15], while others have noted no change in functional scores [6]. Limb salvage with adjuvant radiation therapy of upper extremity sarcomas results in 85–95% sustained local control, and 5-year survivals of 75–80% [1]. A summary of findings from the literature can be found in Table 1.

**Table 1.** Literature overview.

Title	Authors	Number of cases	Treatment modalities	Mean follow-up (years)	Oncologic outcome
Amputation for extremity soft tissue sarcoma does not increase overall survival: A retrospective cohort study	Alamanda et al.	278	LSS vs. amputation	3.1	No difference between mortality, distant metastases, and local recurrence
Long-term outcome after local recurrence of soft tissue sarcoma: a retrospective analysis of factors predictive of survival in 135 patients with locally recurrent soft tissue sarcoma	Daigeler et al.	135	LSS with or without adjuvant chemo and/or radiation	12.3	Significant prognostic indicators for post-resection survival were histologic grade, tumor site, time to initial recurrence, the number of recurrences, and the surgical margin status attained at the last resection.
Neoadjuvant Chemotherapy and Radiotherapy for Large Extremity Soft Tissue Sarcomas	DeLaney et al.	48	Adjuvant chemo and radiation vs. no adjuvant treatment	4	Gain in disease-free and overall survival compared with a historical control group
Recurrent aggressive chondrosarcoma of the middle phalanx of the index finger: excision and reconstruction with an osteocartilaginous allograft	Exner et al.	1	LSS	12	Digit-sparing techniques may be considered rather than ablative procedures
A randomized phase II study on neo-adjuvant chemotherapy for ‘high-risk’ adult soft tissue sarcoma	Gortzak et al.	134	Amputation or LSS with or without chemotherapy	7.3	Neo-adjuvant chemotherapy does not negatively affect the ability to perform surgery
Neo-adjuvant chemotherapy alone or with regional hyperthermia for localized high-risk soft tissue sarcoma: a randomized phase III multicenter study	Issels et al.	341	Neoadjuvant chemotherapy with or without regional hypothermia	2.8	Regional hyperthermia increases the benefit of chemotherapy
Squamous Cell Carcinoma of the Skin of the Trunk and Limbs: The Incidence of Metastases and Their Outcome	Joseph et al.	695	LSS or amputation	4	Risk factors associated with the development of metastatic disease were: delayed presentation: large neglected lesions: misdiagnosis; and multiple treatments to the primary lesion
Limb Salvage Surgery and Adjuvant Radiotherapy for Soft Tissue Sarcomas of the Forearm and Hand	Bray et al.	25	LSS or amputation	3.1	Limb salvage surgery, with adjuvant radiotherapy when necessary, is an effective alternative to amputation in the majority of patients with sarcoma of the forearm and hand.
Localized Operable Soft Tissue Sarcoma of the Upper Extremity	Collin et al.	108	LSS or amputation	8.2	Predictors of local failure: presentation with local recurrence, surgery by LSS, inadequate margins, angiosarcoma, and invasion of vital structures.
Primary reconstruction with digital ray transposition after resection of malignant tumor	Muramatsu et al.	4	Digital ray transposition after tumor resection	6.9	Primary reconstruction with digital ray trans position produces acceptable functional outcomes after resection of malignant tumor.
Preoperative versus postoperative radiotherapy in soft tissue sarcoma of the limbs: a randomized trial	O’Sullivan et al.	190	Preoperative radiation vs. postoperative radiation	3.3	Choice of regimen for patients with soft tissue sarcoma should take into account the timing of surgery and radiotherapy, and the size and anatomical site of the tumor
Outcomes after flap reconstruction for extremity soft tissue sarcoma: A case-control study using propensity score analysis	Kang et al.	148	Flap reconstruction vs. primary closure	5.4	Flap reconstruction had increased morbidity associated with flap reconstruction, but better local control, when compared to patients with primary closure
Chondrosarcoma of Small Bones of the Hand	Patil et al.	23	Curettage, excision, ray resection/amputation	8.5	Results show a high rate of recurrence following curettage, therefore it cannot be recommended for most patients
Single Ray Amputation for Tumors of the Hand	Puhaindran et al.	25	Ray amputation with or without radiotherapy	3	Single ray amputation for hand tumors has low recurrence rates and high functional scores
Treatment of Soft Tissue Sarcomas of the Extremity	Rosenberg et al.	43	LSS vs. amputation	3	LSS, radiation therapy, and adjuvant chemotherapy are capable of successfully treating the majority of adult patients with soft tissue sarcomas of the extremity
Standardization of rehabilitation after limb salvage surgery for sarcomas improves patients’ outcome	Shehadeh et al.	59	LSS	2	Use of standardized rehabilitation protocol resulted in improved patient functional outcome
Functional and oncological outcomes after limb salvage surgery for primary sarcomas of the upper limb	Wright et al.	72	LSS with or without adjuvant chemotherapy and/or radiotherapy	2.8	Limb salvage surgery is applicable to a wide range of tumor types and grades, to all patient age groups, and anatomical sites with good functional results

* LSS = limb salvage surgery.

## Reconstruction

In terms of maintaining maximal function, limb salvage surgery is advantageous, and may require soft tissue and neurovascular reconstruction. Reconstruction can either be performed at the time of resection or staged after a short interval, usually if there is concern about inadequate margins [16]. Repair warrants important considerations, including defect size, timing of reconstruction, defect location, neurovascular structure, patient functional status, scar contracture, and the benefits of avoiding multiple surgical procedures [10]. Following resection, reconstruction options are many, including primary closure, skin grafting, local soft tissue flaps, regional pedicle and island flaps, free tissue transfer, composite free tissue transfer, allografts, endoprostheses, and tendon, nerve, or arterial grafting [10]. These options are discussed below.

Generally, reconstruction should be attempted in the simplest possible method [11]. The approach to reconstruction generally starts with vascular reconstruction, followed by establishing a stable bony skeleton, followed by reconstruction of critical nerves and tendons [11]. Reconstruction of these structures, when performed immediately following tumor resection, allows for fewer surgeries, earlier mobilization, and a faster recovery [11]. When severing nerves, truncation of nerves proximally helps to avoid neuroma formation at the stump [2].

The most basic option in reconstruction is primary closure, which is highly dependent on the size of the defect created by the resection and limits of tension [10, 11]. Primary closure can be used for small defects, however repair under tension, which is more likely to occur with larger defects, will often result with wound contracture and subsequent decreased functional outcomes [10]. Flaps and skin grafts avoid this problem. Local flaps can mitigate this issue, such as Z-plasty and rhomboid flaps, whose main benefits are to increase length of the scar and decrease tension, respectively [10]. The theory of a Z-plasty is to rotate the axis of contracture away from the plane of maximum tension and lengthens the scar by recruiting local tissue excess [10]. Larger angles used in Z-plasties lead to larger gains in length at the expense of tension [10]. With skin grafting in relation to the hand, full-thickness grafts are preferred due to increased limits of wear and decreased rates of contraction [10] as well as improved cosmetic outcomes [11]. Rotational flaps allow spreading of tension over a larger area [10]. Advancement flaps are often used in fingertip amputations or on the dorsum of the hand, and given their local nature, result in excellent color and texture matching [10]. Free flaps are useful when skin grafting or local flaps would cause undesirable results. Disadvantages of this approach include the possibility of flap failure and the prolonged operating times [10]. Commonly used free flaps include the rectus free flap, scapular free flap, latissimus dorsi free flap, gracilis free flap, and fibula free flap [10]. The fibula free flap is useful when significant amounts of vascularized bone are required for reconstruction [10]. Caution must be exercised when proceeding with complicated coverage and flaps, as this adds stress on the blood supply to an area which may require radiation, thus impeding healing [2]. The most important factor in flap survival is surgical experience [10]. Pedicle flaps warrant careful consideration in oncologic cases given the concerns for tumor seeding adjacent regions resulting in local recurrence [1, 2].

**Figure 1.**
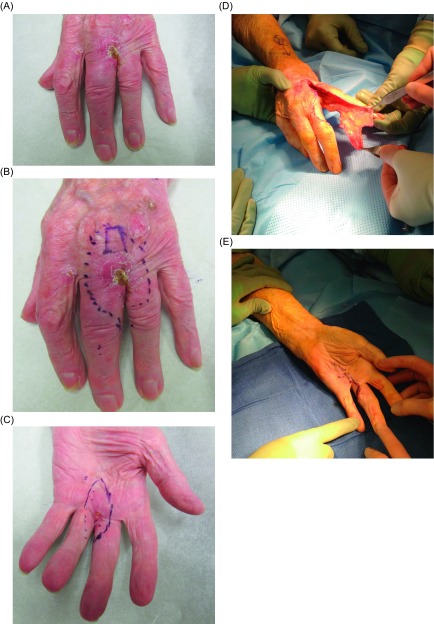
(A) Squamous cell carcinoma of the left hand near the interdigital web space between the third and fourth metacarpals. (B, C) Preoperative markings at volar and dorsal aspects. (D) Intraoperative ray resection with filet flap preserved from disease-free zone for coverage of soft tissue defect. (E) After ray resection and incision closure with filet flap.

Partial hand amputations, such as single or double ray amputations, can provide oncologic cure while maintaining good functional outcomes [15]. Ray amputations (Figure 1) are often necessary to achieve negative margins in the management of sarcomas of the hand [4]. In addition to adequate control, ray amputations also provide acceptable functional outcomes, however emotional acceptance of a three-fingered hand may be deemed unsatisfactory by some patients [17]. Given the specialized nature of palmar skin, reconstruction is particularly challenging [10]. The dorsum of the hand is more forgiving given the axial blood supply and loose skin, thus allowing moderately sized defects to be closed primarily [10]. In digit reconstruction, goals include maintenance of sensation, length, and flexibility [10]. In these instances, defects as large at 1 cm undergo satisfactory healing by secondary intention [10]. Skin grafts can be used for both temporary and definitive treatment options, however with a major drawback of lack of sensation [10]. In soft tissue sarcomas of the distal digits, adequate surgical margins are usually obtained by disarticulations at the distal interphalangeal joint (DIP) or proximal interphalangeal (PIP) joints [1].

Limb salvage often creates complex soft tissue defects that are prone to difficulties in wound healing [9]. It is generally assumed that patients undergoing flap reconstruction, which is considered to be the most complex form of reconstruction, will have increased morbidity given the added complexity of this treatment modality [9]. This assumption is based on the hurdles, such as vascular vulnerability and donor/recipient site morbidity [9], brought about by flap reconstruction. However, there is conflicting data regarding the impact of flap reconstruction on the rate of complications following limb salvage surgery [9]. Interestingly, patients with soft tissue sarcoma of the extremities were found to have lower recurrence rates with flap reconstruction than primarily closure, which may be attributable to wider surgical margins used with flap reconstruction in comparison to primary closure [9].

## Radiation

Adjuvant radiation, although effective in improving local control after resection, is not necessary or necessarily beneficial in all patients [5]. Radiotherapy is less likely to be necessary in those patients with small low-grade lesions that are resected with widely negative margins [5]. Benefits of radiation are more likely to be appreciated when patients present with high-grade lesions, large lesions, or borderline or positive margins [5]. In cases where limb salvage and external beam radiation are used, the benefits of preoperative versus postoperative radiation have been debated [1, 18]. Preoperative radiation has a few benefits: first, there is a higher rate of margin-negative resection in large tumors or those in close proximity to vital structures [5]. This is due to tumor necrosis which reduces tumor size and resultant tumor contamination during surgery [1]. Second, the doses used tend to be lower and radiation fields smaller, thus limiting damage to normal tissue [1, 5, 18]. Overall, these factors result in lower long-term toxicity seen with the preoperative approach [5]. The disadvantages of the preoperative approach include higher wound complication rates [18], with one study finding quadruple the wound complication rate [19]. As a result, preoperative radiation is primarily reserved for those whose tumor location is prohibitive of wide resection techniques, such as those near neurovascular structures [1]. Despite the theoretical advantages and disadvantages, no survival benefit has been demonstrated between preoperative and postoperative external beam radiation [1, 18].

Attempting to preserve important neurovascular and other functional structures in limb-sparing procedures may compromise the adequacy of surgical margins, resulting in increased risk of local recurrence [4]. Previous radiotherapy to a recipient or donor site in flap procedures may influence vascular availability [10]. Invasion into critical neurovascular structures complicates limb salvage and often leads to poorer outcomes [8] as well as higher rates of recurrence [7]. Healing by secondary intention when adjuvant radiation is used is more likely to result in chronic non-healing wounds [2]. Radiation exposure to adjacent normal tissue can be further decreased with brachytherapy [5]. Postoperative brachytherapy, through sparing of adjacent normal tissue, results in decreased complications such as edema and stiffness, with delayed initiation of radiation further lowering complication rates [1]. Perioperative brachytherapy results in better local control than surgery alone with high-grade lesions [16].

## Chemotherapy

The use of adjuvant chemotherapy remains controversial [1, 5]. The recurrence rates with adjuvant chemotherapy are reduced, however the presence and duration of benefits remains unsettled [5]. Neoadjuvant chemotherapy provides the theoretical advantages of control of micrometastatic disease as well as facilitating resection of the primary tumor [20]. Those with high-risk metastatic disease, including large, high-grade, and deep lesions, are more likely to benefit from chemotherapy [5]. More favorable results have been reported with neoadjuvant use of certain agents, especially when combined with radiation therapy [1]. DeLaney found improved 5-year survival using preoperative mesna, adriamycin, ifosfamide, and dacarbazine (MAID therapy) combined with radiation therapy compared to postoperative radiation therapy alone [20]. A separate randomized phase II study was unable to demonstrate survival benefit with neo-adjuvant chemotherapy after seven years [21].

Additional therapies, such as regional hyperthermia, can be used as both a radiosensitizer and a chemosensitizer by causing direct thermal toxicity, increasing drug efficacy, and inducing tumoricidal immune responses [22, 23]. In combination with neo-adjuvant chemotherapy, it has been demonstrated to increase benefits of neo-adjuvant chemotherapy [23].

## Oncological subtypes

The most common subtypes of sarcoma in the upper extremity include synovial sarcomas, epithelioid sarcomas, clear cell sarcomas, and malignant fibrous histiocytomas [1, 5]. Extremity sarcomas generally present with localized disease, however 10% present with metastatic involvement which is primarily in the lung, lymph nodes, and bone [5]. Factors that increase the risk of metastases include larger size, deep to superficial fascia, high-grade tumors, and high-risk histologic subtypes [5]. Recurrence in extremity locations has a significantly better prognosis compared to non-extremity lesions [24]. Other factors in recurrent disease associated with improved survival include late recurrence and low histologic grade [24]. In recurrent disease, the effect of adequate versus inadequate surgical margins has increased impact [24]. Cases in which re-excision was performed resulted in poorer outcomes compared to those that underwent primary wide resections [4]. It is important to note that in metastatic disease, malignant cells invade the blood vessels to reach distant site [8]. In these cases, amputation will control local disease, however it is unable to change the course of these malignant cells when migration has already taken place [8]. The site of biopsy becomes very important, as the needle can seed tumor cells contributing to local recurrence if the biopsy site and tract is not excised during definitive surgery [5]. Additionally, biopsy incisions should be placed longitudinally to better accommodate proper resection and minimal violation of surrounding normal tissues [1, 5].

Overall survival and long-term outcomes of sarcomas are dependent on histologic grade, depth, tumor size, and histologic subtype [24]. Tumor size is an important factor in predicting metastasis-free survival and overall survival [5]. Often distal masses are identified sooner and are therefore smaller on presentation [5]. Those tumors which lie deep have a worse prognosis than superficial tumors [5]. As expected, local treatment failure rates increase as the margin size decreases [7]. In fact, the presence of positive margins is the most important factor in predicting recurrence [5]. However, it has been suggested that rather than positive margin status, the inherent aggressiveness of the tumor is more indicative of the final outcome, meaning that margin status is a result of biological aggressiveness rather than a cause [24]. In accordance with this, Daigeler found similar survival between certain individuals with residual tumor compared to those with complete resection and negative margins [24]. Based on these findings, it seems negative margins provide long-term benefit in patients with locally recurrent soft tissue sarcomas, however achievement of negative margins at all costs (including amputation in cases where salvage is not possible) may not be necessary and should be tailored to individual cases [24].

Chondrosarcoma accounts for about 4% of hand tumors [25, 26] and 40% of malignant bone tumors of the hand, yet despite their rare occurrence, they are the most common malignant bone tumor in the hand [3, 11]. Primary chondrosarcoma of the hand and wrist usually arises de novo, however malignant degeneration of a preexisting lesion, such as those with enchondromatosis or osteochondromatosis, may occur as well [3, 27].

The optimal treatment approach of chondrosarcoma is not clear based on differing information on recurrence rates. Chondrosarcoma of the hand is usually aggressive and high-grade, however the phalangeal form is characterized by local recurrence [25] and minimal metastatic potential in contrast to chondrosarcomas located elsewhere [3]. The reason for this variable behavior has been postulated to be due to the small size of hand tumors, low temperature in the digits, or differences in tumor development [26]. Despite the more benign nature of chondrosarcomas in the hand, optimal treatment is not clear.

**Figure 2.**
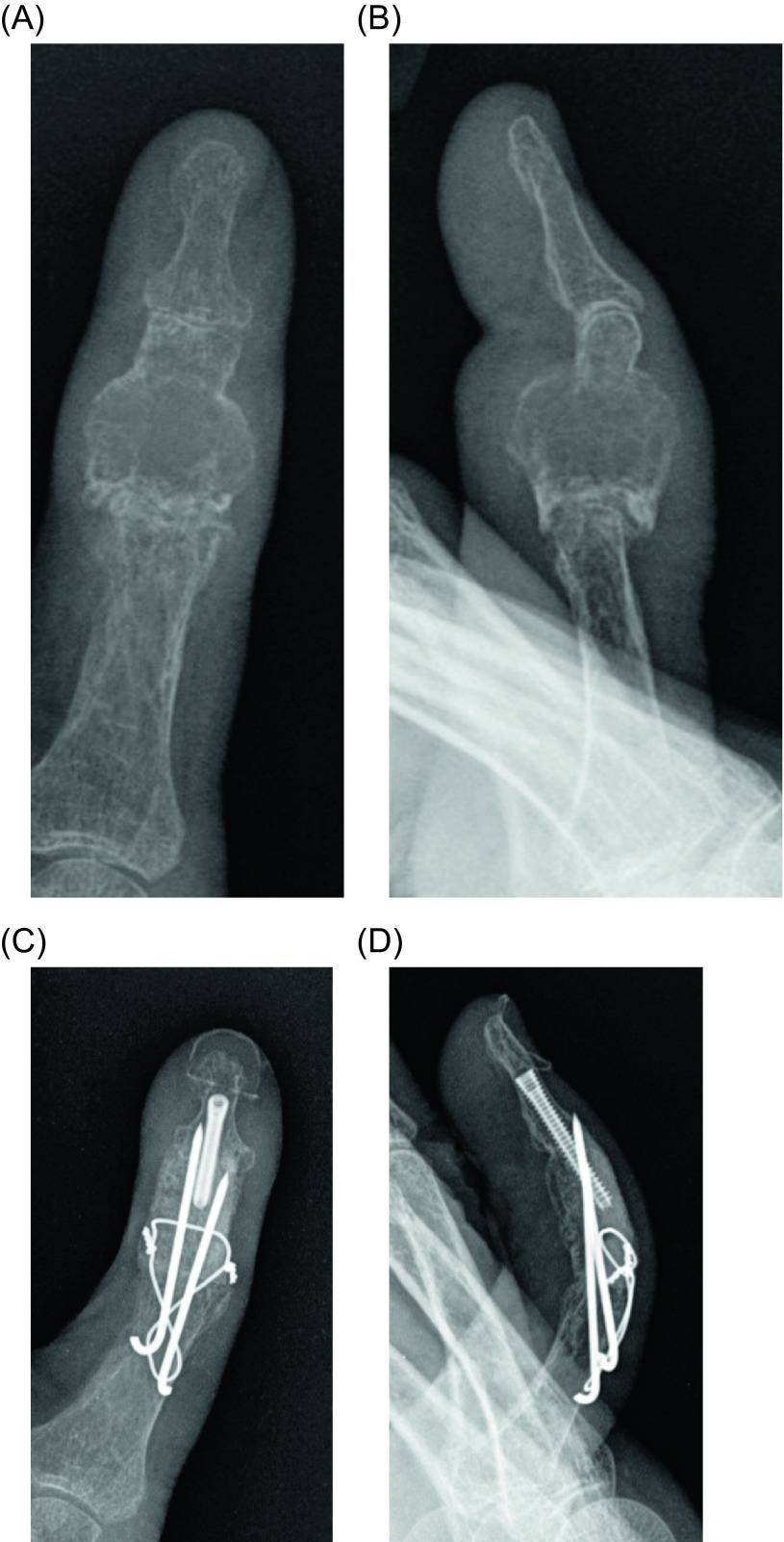
(A) Chondrosarcoma of the left hand at the second metacarpal and proximal phalanx. (B) Status post-ray amputation of the involved index finger with preservation of a filet flap from the distal uninvolved tissue from the amputated index finger to (C) cover the soft tissue defect after tumor resection.

**Figure 3.**
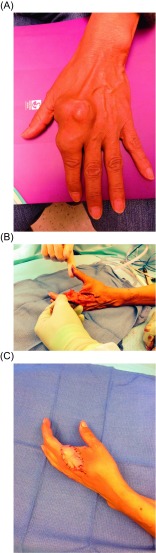
(A) Posterior-anterior (PA) and (B) oblique views of radiograph demonstrating chondrosarcoma at the thumb proximal phalanx. (C) PA and (B) oblique views postoperatively after resection and limb salvage surgery (LSS) reconstruction.

Traditional advice for treatment has gravitated toward ray resection or digital amputation with wide margins to prevent local recurrences (Figure 2) [26]. Treatment of chondrosarcoma was previously wide en bloc excision either through limb salvage or amputation [27], however recent literature advocates intralesional excision with close follow-up [3]. Amputation or ray resection results in excellent local control, while a significant rate of local recurrence has been reported in patients with curettage or local excision [27]. Due to its more benign nature, some researchers suggest that a more conservative surgical approach is warranted to preserve function (Figure 3) [25, 28]. This approach is supported by similar survival rates between both amputation and curettage with adequate follow-up, thus suggesting that limb-sparing procedures should take precedence, especially in cases where amputation would lead to a significant loss of hand function [29]. However, others suggest the more traditional approach of ray resection or digital amputation, except in exceptional circumstances such as old and frail patients, due to other differing results demonstrating a higher rate of recurrence following curettage [26].

Chondrosarcomas of the phalanges are generally less aggressive than chondrosarcomas located to the metacarpals and metatarsals [28]. In those patients with high risk for local recurrence, reconstruction using total allograft replacement of the phalanx is an important option to consider, as removal of the total bone offers a higher chance of cure [28].

## Summary

In the treatment of upper extremity sarcoma, the main treatment distinction is between limb salvage and amputation. Given the detrimental effects on function and psychologic outcomes, amputation has been superseded by limb salvage in most cases, although it can occasionally be the only option. Radiation therapy reduces recurrence, although preoperative and postoperative radiation carry unique benefits and risks. Brachytherapy is beneficial to patients with high-grade lesions. The use of chemotherapy outside of certain subtypes remains controversial. Regional hyperthermia is an emerging therapy which appears beneficial as a radiosensitizer and chemosensitizer, although further investigation is warranted.

## Conflict of interest

KJ certifies that he or she has no financial conflict of interest (e.g., consultancies, stock ownership, equity interest, patent/licensing arrangements, etc.) in connection with this article.

GD certifies that he or she has no financial conflict of interest (e.g., consultancies, stock ownership, equity interest, patent/licensing arrangements, etc.) in connection with this article.

RA certifies that he or she has no financial conflict of interest (e.g., consultancies, stock ownership, equity interest, patent/licensing arrangements, etc.) in connection with this article.

AK certifies that he or she has no financial conflict of interest (e.g., consultancies, stock ownership, equity interest, patent/licensing arrangements, etc.) in connection with this article.
